# Supplementary material for: Primordial mimicry induces morphological change in Escherichia coli
Source: Commun Biol. 2022 Jan 11;5:24. doi: 10.1038/s42003-021-02954-w (PMC8752768; doi:10.1038/s42003-021-02954-w)
Supplement: Supplementary file 2 — Supplementary information S1-S12 [file 42003_2021_2954_MOESM2_ESM.pdf]

Supplementary information

Primordial mimicry induces morphological change in *Escherichia coli*

Hui Lu[1], Masaomi Kurokawa[2], Honoka Aita[2], Feng Chen[3], Yang Xia[1], Jian Xu[1], Kai Li[1], Bei-Wen Ying[2,*], Tetsuya Yomo[1,*]

[1]Biomedical Synthetic Biology Research Center, School of Life Sciences, East China Normal University, 3663 North Zhongshan Road, Shanghai 200062, PR China

[2]Graduate School of Life and Environmental Sciences, University of Tsukuba, 1-1-1 Tennoudai, Tsukuba, Ibaraki 305-8572, Japan

[3]School of Software Engineering, East China Normal University, 3663 North Zhongshan Road, Shanghai 200062, PR China

[*]Correspondence: ying.beiwen.gf@u.tsukuba.ac.jp (BW.Y.), tetsuyayomo@gmail.com (T.Y.)

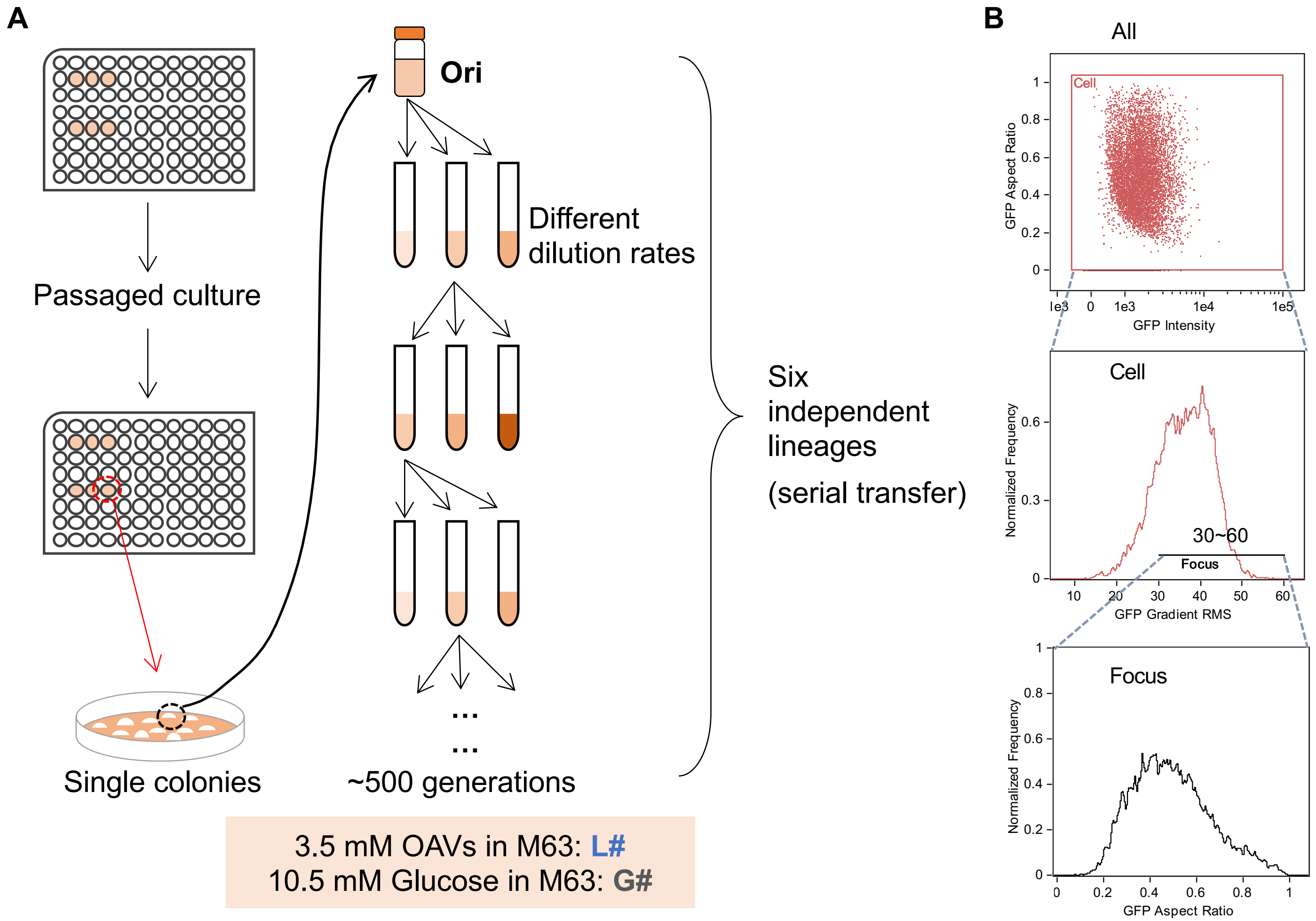

Supplementary Figure 1. Schematic drawing of the experimental evolution (A) and imaging flow cytometry gating strategy (B).

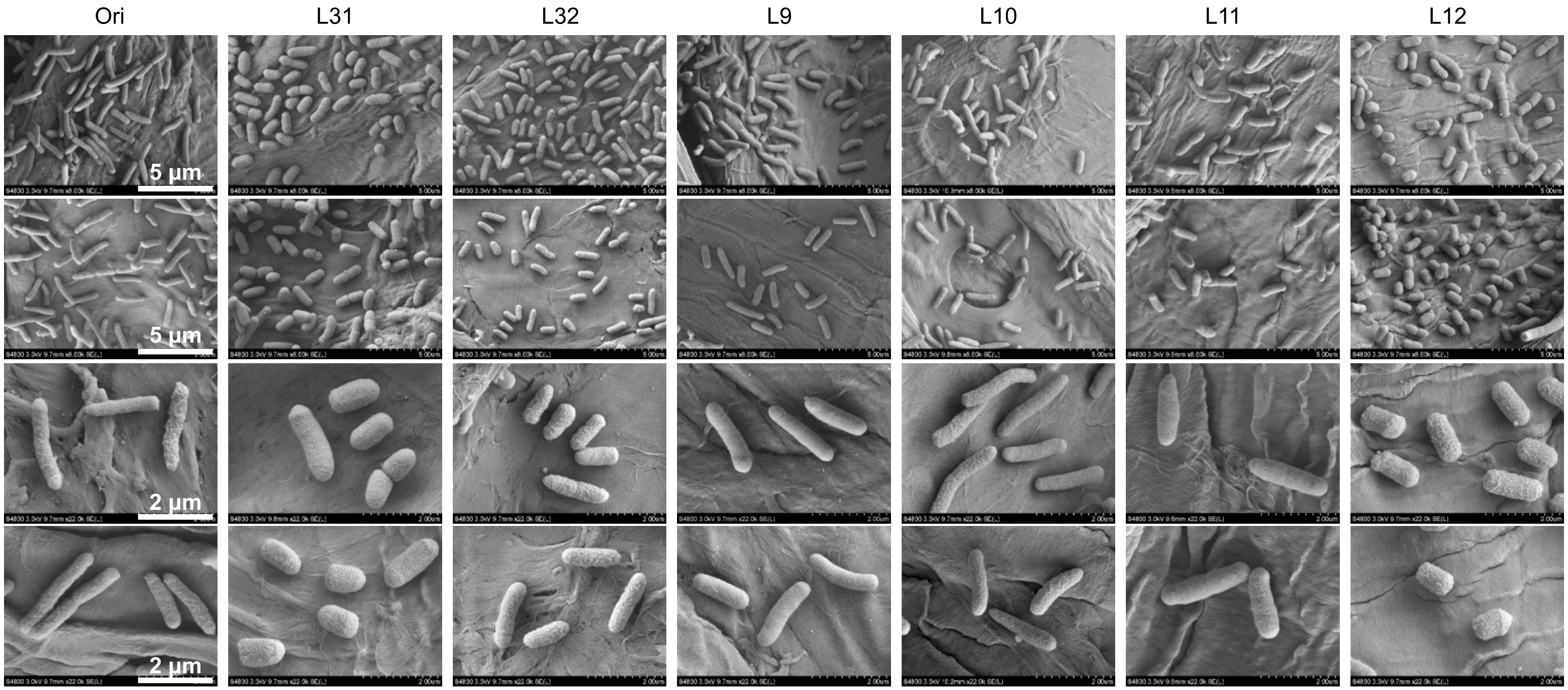

Supplementary Figure 2. Cell shapes imaged by SEM. Additional single-cell images of the Ori and the six lineages evolved in OAVs are shown on two size scales. Scale bars are indicated.

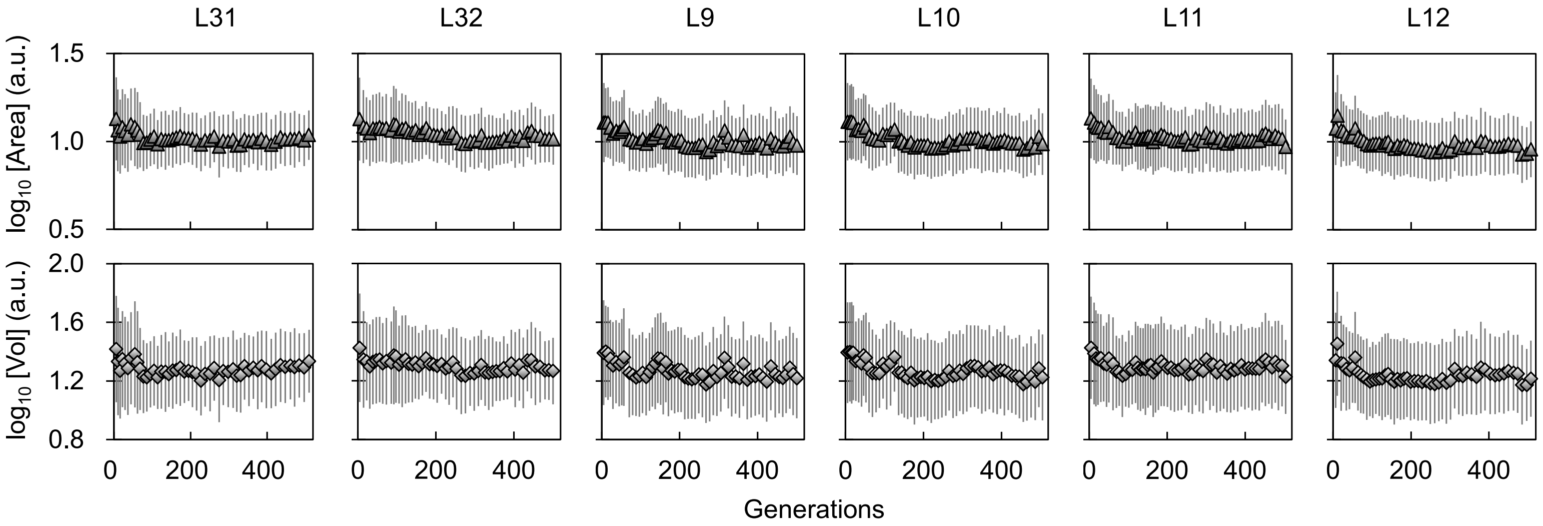

Supplementary Figure 3. Changes in cell size during experimental evolution. Changes in the cell size, represented by the relative area (**A**) and the relative volume (**B**), are shown. The means and standard deviations of the cell populations are represented by horizontal and vertical black lines, respectively. The six evolutionary lineages are indicated.

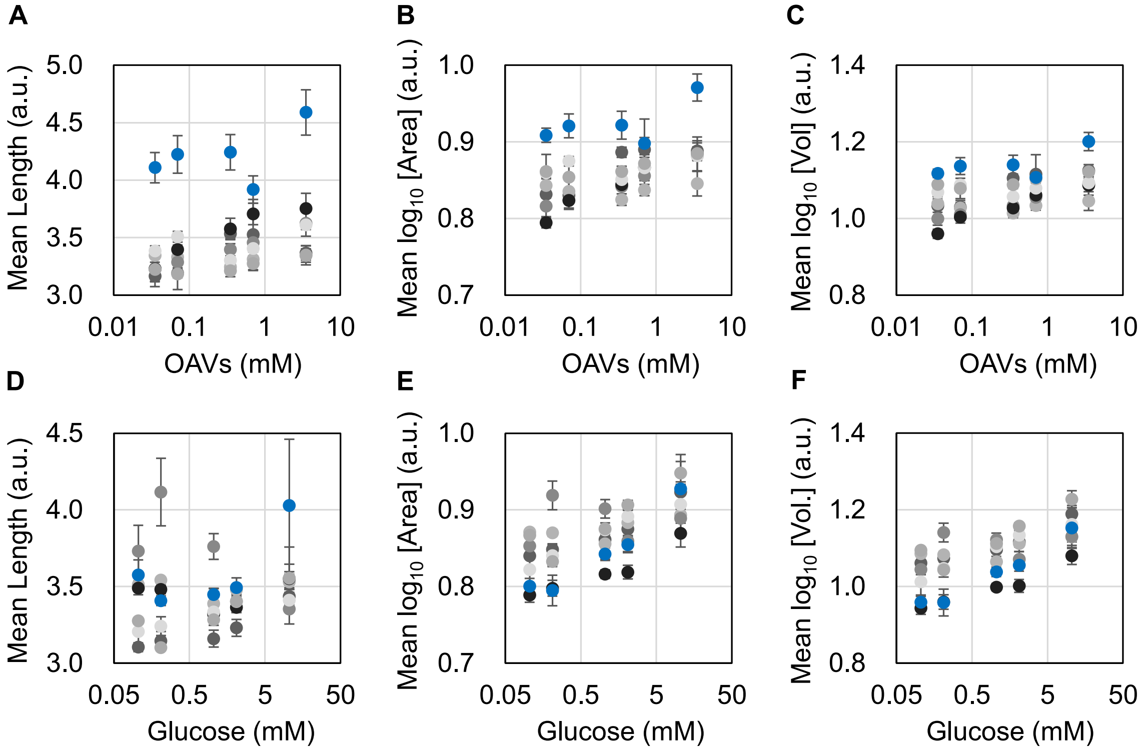

Supplementary Figure 4. Cell morphology in OAVs and glucose. The steady states of the cell shapes and sizes in the presence of OAVs (**A**~**C**) or glucose (**D**~**F**) are shown. The averages of the mean cell length and the mean logarithmic area and volume of the cell populations are indicated. The Ori and Evos are indicated as blue and colourless circles, respectively. The grey circles (gradation from light to dark grey) indicate the six lineages. Standard errors of biological replications (N>5) are indicated.

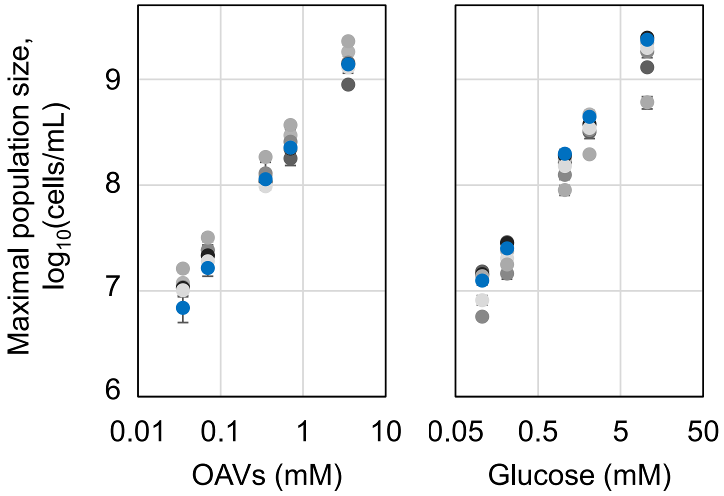

Supplementary Figure 5. Maximal population densities in OAVs and glucose. The saturated cell populations grown in the presence of varied concentrations of carbon sources were measured. The left and right panels indicate the carbon sources OAVs and glucose, respectively. The Ori and Evos are indicated as blue and colourless circles, respectively. The grey circles (gradation from light to dark grey) indicate the six lineages. Standard errors of biological replications (N>5) are indicated.

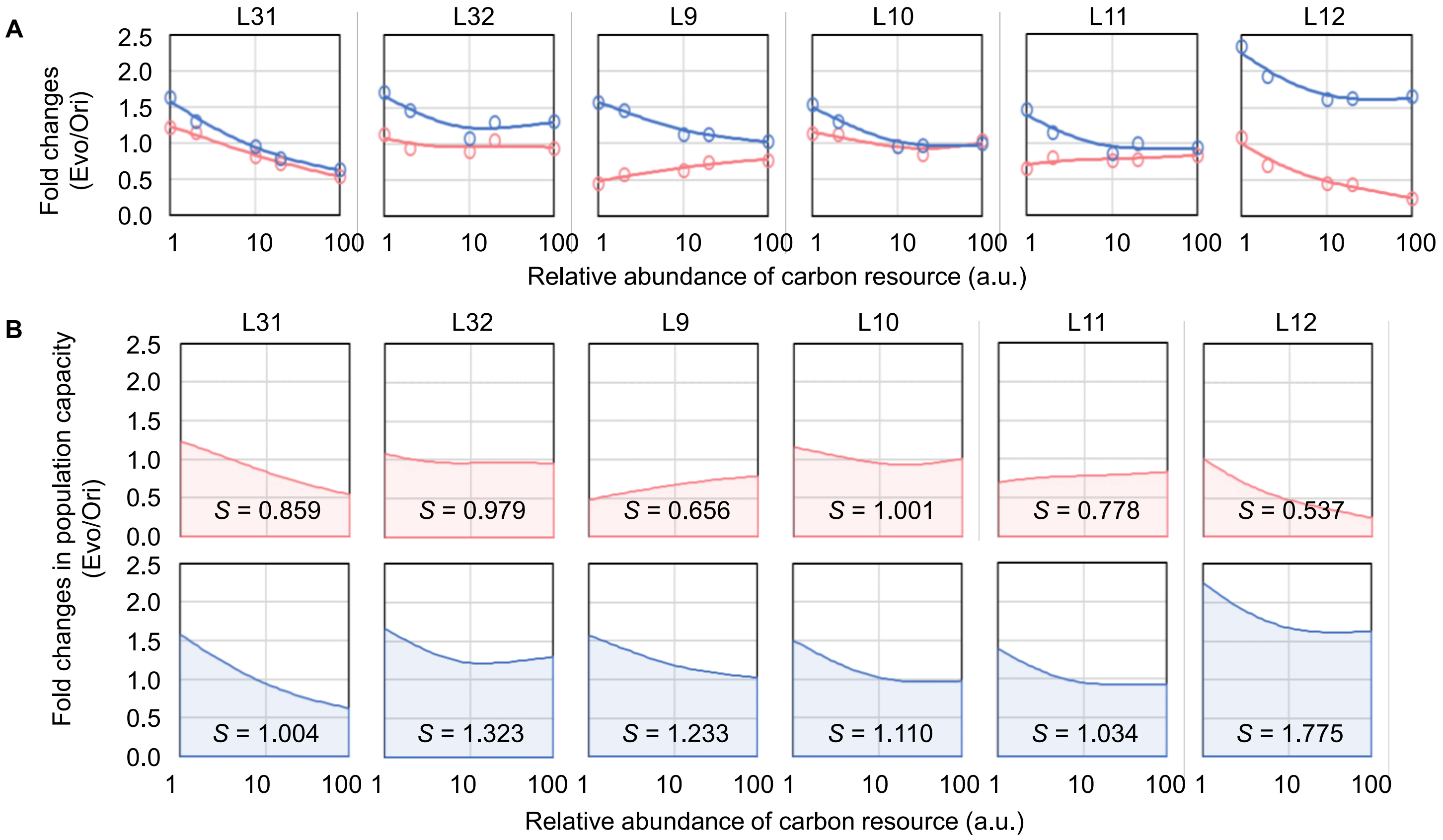

Supplementary Figure 6. Fold changes in population capacity. A. Theoretical fitting (spline curve) of the fold changes in carbon utilization. The fold changes in the carrying capacity of the Evos relative to that of the Ori were calculated and plotted against the normalized carbon amount. Fitted curves (solid lines) with their experimental data points (open circles) are shown. Blue and red indicate the carrying capacities in medium supplemented with OAVs and glucose, respectively. **B.** Fitted areas of the fold changes in carrying capacity. The upper and bottom panels represent glucose and OAVs, respectively. *S* indicates the relative value of the fitted area.

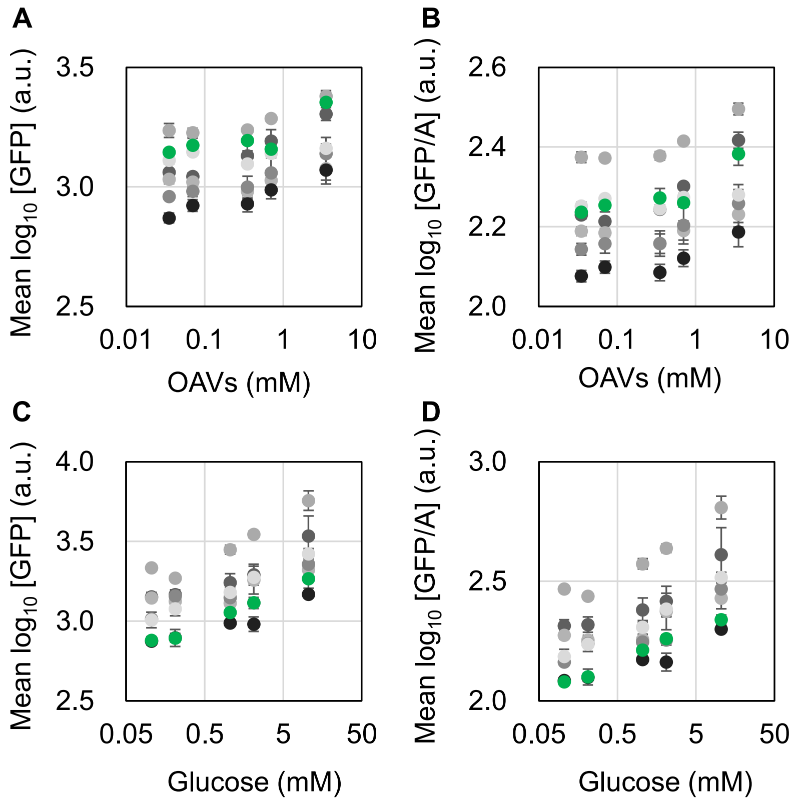

Supplementary Figure 7. Total amounts and relative abundances of cellular protein. The total amounts and relative abundances of cellular protein in the presence of OAVs (**A-B**) and glucose (**C-D**) are represented by GFP and GFP/A, respectively. The Ori and Evos are indicated as green and colourless circles, respectively. The grey circles (gradation from light to dark grey) indicate the six lineages. Standard errors of biological replications (N>5) are indicated.

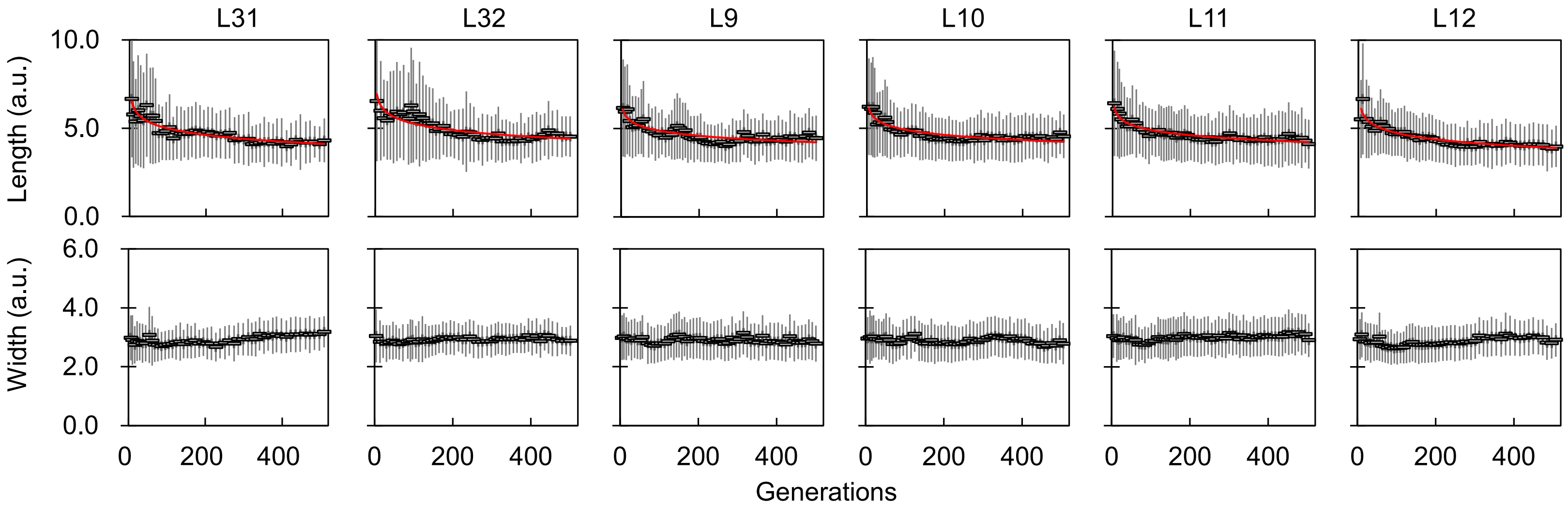

Supplementary Figure 8. Changes in cell length and width during experimental evolution. Changes in the cell length (upper panels) and width (bottom panels) are shown. The means and standard deviations of the cell populations are represented by horizontal and vertical black lines, respectively. The six evolutionary lineages are indicated. Logarithmic regression of the temporal changes is represented by the red solid curve.

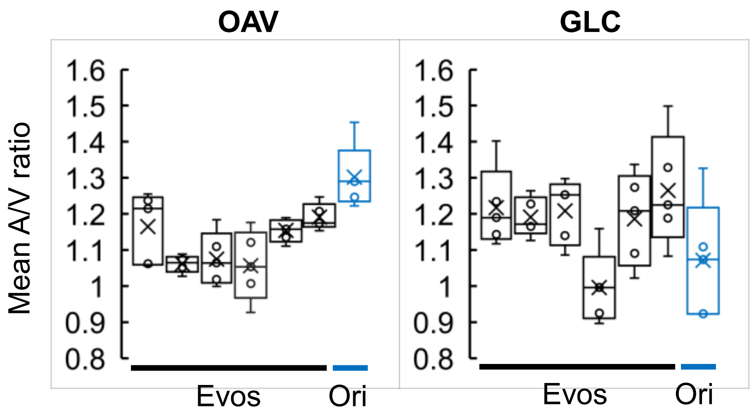

Supplementary Figure 9. Boxplots of the area-to-volume ratios for various carbon sources. The Ori (blue) and the six Evos L31, L32, L9, L10, L11, and L12 (from left to right, black) were grown in the presence of either OAVs (left panel) or glucose (right panel). The mean area-to-volume (A/V) ratios of the cell populations (measurements) are indicated as circles. The medium and the average of the mean A/V ratios are represented as lines and crosses inside the box, respectively.

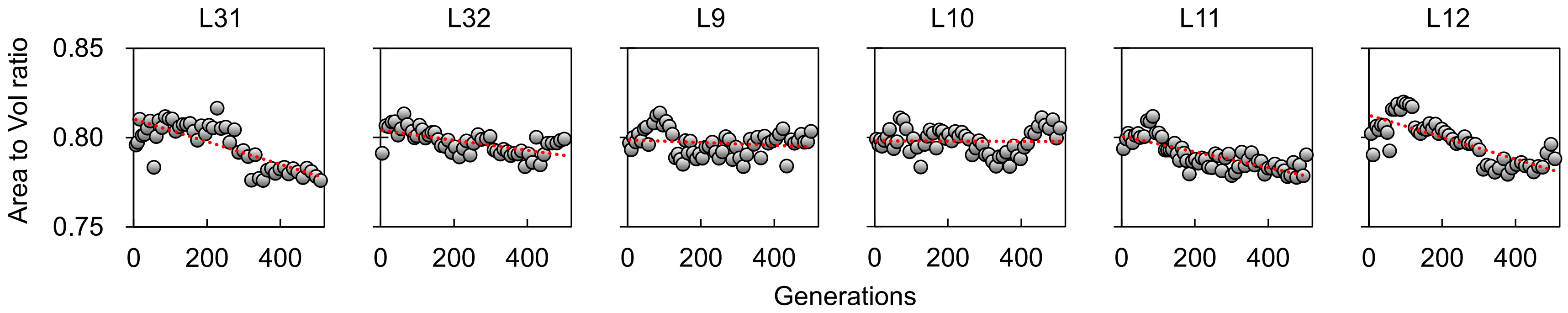

Supplementary Figure 10. Evolutionary changes in the area-to-volume ratio. The area-to-volume ratio was calculated by dividing the mean area by the mean volume of the cell population. A linear regression of the changes is indicated by the red broken line. The six evolutionary lineages are indicated.

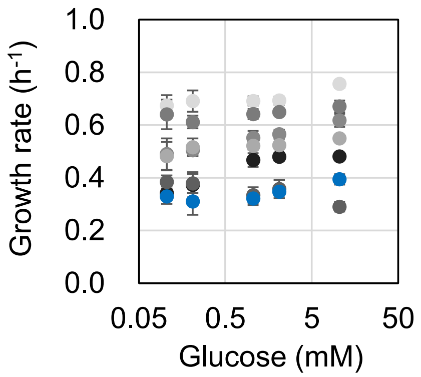

Supplementary Figure 11. Growth rates in glucose. The growth rates of Ori and Evos in minimal medium supplemented with various concentrations of glucose are indicated as blue and colourless circles, respectively. The grey circles (gradation from light to dark grey) indicate the six lineages. Standard errors of biological replications (N>5) are indicated.

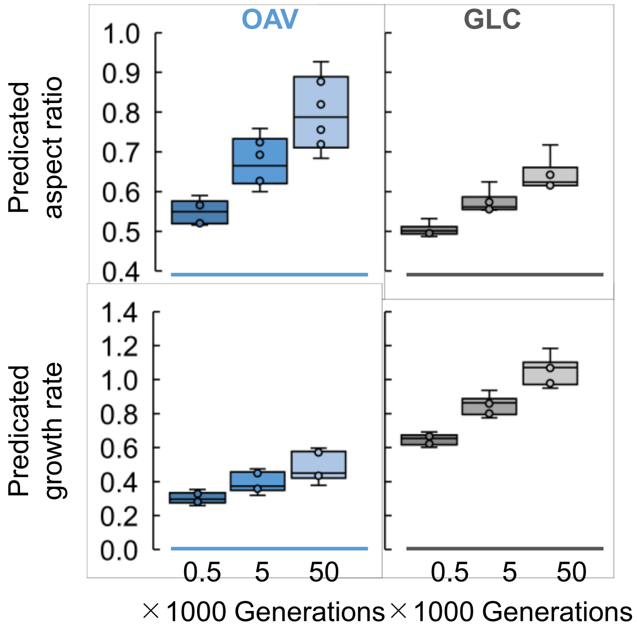

Supplementary Figure 12. Theoretical prediction of the growth fitness and cell shape. The growth rates and the aspect ratios of the *E. coli* cells experienced 500, 5,000 and 50,000 generations in OAVs (left) or glucose (right) were estimated, according to the logarithmic regression of the six evolutionary lineages shown in Fig. 1.
