# Supplementary material for: Primordial mimicry induces morphological change in Escherichia coli
Source: Commun Biol. 2022 Jan 11;5:24. doi: 10.1038/s42003-021-02954-w (PMC8752768; doi:10.1038/s42003-021-02954-w)
Supplement: Supplementary file 3 — Description of Additional Supplementary Files [file 42003_2021_2954_MOESM3_ESM.pdf]

Description of Additional Supplementary Files

File name: Supplementary Data 1

Description: Growth rate and aspect ratio acquired in the transfer to OAVs and glucose. The generation passaged number, growth rate and aspect ratio of the E. coli population selected for the daily transfer are indicated as Gen., Rate and A.R., respectively. The evolutionary lineages in OAVs or glucose are represented by L# or G#, respectively.

File name: Supplementary Data 2

Description: Correlation analyses of the evolutionary lineages in OAVs. Spearman correlation coefficients and the statistical significance of all features acquired with the imaging flow cytometer are shown. The Spearman rank correlation coefficients and the significance are indicated as cor and p, respectively. Asterisks represent high significance.

File name: Supplementary Data 3

Description: Correlation analyses of the evolutionary lineages in glucose. Spearman correlation coefficients and the statistical significance of all features acquired with the imaging flow cytometer are shown. The Spearman rank correlation coefficients and the significance are indicated as cor and p, respectively. Asterisks represent high significance.

File name: Supplementary Data 4

Description: Genome mutations fixed in the six lineages. The detected mutations with frequencies higher than 60% in the endpoint populations are summarized. The gene name, essentiality, chromosomal position, ratio of mutant in the population (frequency), changes in nucleotide and amino acid, related protein domain, reference (Pfam) and function are indicated. n.d. stands for not determined.

File name: Supplementary Data 5

Description: All mutations detected in the experimental evolution. The gene name, essentiality, chromosomal position, changes in nucleotides and amino acids and function are indicated. In addition, the ratio of mutants in the intermediate (~300 generations) and endpoint (~500 generations) populations are represented by Frequency.

File name: Supplementary Data 6

Description: Data summary of resource utilization. Population represents the six Evos and Ori. Resource type and Resource conc indicate the carbon source and the corresponding concentration used in the assay, respectively. The data of mean Aspect ratio, population capacity (cells/mL/mM) and mean log10(GFP/V) are shown, which are used to create Fig. 4 and Fig. 5.
