# Supplementary material for: Primordial mimicry induces morphological change in Escherichia coli
Source: Commun Biol. 2022 Jan 11;5:24. doi: 10.1038/s42003-021-02954-w (PMC8752768; doi:10.1038/s42003-021-02954-w)
Supplement: Supplementary file 10 — Reporting Summary [file 42003_2021_2954_MOESM10_ESM.pdf]

nature research

Corresponding author(s): Tetsuya Yomo

Last updated by author(s): Oct 23, 2021

Reporting Summary

Nature Research wishes to improve the reproducibility of the work that we publish. This form provides structure for consistency and transparency in reporting. For further information on Nature Research policies, see our Editorial Policies and the Editorial Policy Checklist.

Statistics

For all statistical analyses, confirm that the following items are present in the figure legend, table legend, main text, or Methods section.

n/a	Confirmed	
☐	☒	The exact sample size (*n*) for each experimental group/condition, given as a discrete number and unit of measurement
☐	☒	A statement on whether measurements were taken from distinct samples or whether the same sample was measured repeatedly
☐	☒	The statistical test(s) used AND whether they are one- or two-sided<br>*Only common tests should be described solely by name; describe more complex techniques in the Methods section.*
☐	☒	A description of all covariates tested
☐	☒	A description of any assumptions or corrections, such as tests of normality and adjustment for multiple comparisons
☐	☒	A full description of the statistical parameters including central tendency (e.g. means) or other basic estimates (e.g. regression coefficient) AND variation (e.g. standard deviation) or associated estimates of uncertainty (e.g. confidence intervals)
☐	☒	For null hypothesis testing, the test statistic (e.g. *F*, *t*, *r*) with confidence intervals, effect sizes, degrees of freedom and *P* value noted<br>*Give P values as exact values whenever suitable.*
☒	☐	For Bayesian analysis, information on the choice of priors and Markov chain Monte Carlo settings
☒	☐	For hierarchical and complex designs, identification of the appropriate level for tests and full reporting of outcomes
☐	☒	Estimates of effect sizes (e.g. Cohen's *d*, Pearson's *r*), indicating how they were calculated

Our web collection on statistics for biologists contains articles on many of the points above.

Software and code

Policy information about availability of computer code

Data collection	Microsoft Excel, Gen5 3.05, IDEAS software v.6.2.183.0,
Data analysis	Microsoft Excel, IDEAS software v.6.2.183.0, Python3, Genome Analysis Toolkit (GATK)

For manuscripts utilizing custom algorithms or software that are central to the research but not yet described in published literature, software must be made available to editors and reviewers. We strongly encourage code deposition in a community repository (e.g. GitHub). See the Nature Research guidelines for submitting code & software for further information.

Data

Policy information about availability of data

All manuscripts must include a data availability statement. This statement should provide the following information, where applicable:

- Accession codes, unique identifiers, or web links for publicly available datasets
- A list of figures that have associated raw data
- A description of any restrictions on data availability

https://dataview.ncbi.nlm.nih.gov/object/PRJNA693085

Field-specific reporting

Ecological, evolutionary & environmental sciences study design

All studies must disclose on these points even when the disclosure is negative.

Study description	Six evolutionary lineages (L31, 32, 9~12) were generated under glucose-free but oleic acid vesicle (OAV)-rich conditions, which were considered as the laboratory primordial environments. Parallel experiments (G31, 32, 9~12) were conducted in the glucose only minimal medium.
Research sample	The E. coli MDS42ΔgalK::Ptet-gfp-kan was used for the experimental evolution. Firstly, cells passaged several times were subjected to single colony isolation by plating the cell culture on LB agar plates, and only one of the single colonies was selected as the Ori for the experimental evolution. Then, each six independent evolutionary lineages were generated from the common stock of the Ori.
Sampling strategy	As for evolutionary experiments, we used six independent samples for overall analysis for comparisons. Based on those strains, we then performed carbon source utilization assays through at least two-independent results. And the growth assays in glucose-supplemented minimal medium were obtained through six replicates for each group.
Data collection	Twelve evolutionary lineages (L31, 32, 9~12; G31, 32, 9~12) were generated from Ori for approximately 500 generations. The E. coli cell populations were analysed using the imaging flow cytometry once or twice a day.
Timing and spatial scale	We transfered E. coli in OAV-supplemented medium in a daily basis until 500 generations. As for the passage in glucose-supplemented medium, twice transfer a day.
Data exclusions	No data were excluded from the analysis.
Reproducibility	To verify the reproducibility of experimental findings, six independent evolutionary lineages (L31, 32, 9~12; G31, 32, 9~12) for each group were generated from the common stock of the Ori .
Randomization	This is not relevant to our study since six independent lineages (L31, 32, 9~12; G31, 32, 9~12) in our study were generated from the common ancestor named Ori. There are no samples allocated into groups.
Blinding	In this study, blinding is not relevant since we determine the specific outcome (e.g. gene mutations and growth curve) from each evolved strains.

Did the study involve field work? ☐ Yes ☒ No

Reporting for specific materials, systems and methods

We require information from authors about some types of materials, experimental systems and methods used in many studies. Here, indicate whether each material, system or method listed is relevant to your study. If you are not sure if a list item applies to your research, read the appropriate section before selecting a response.

Materials & experimental systems

n/a	Involved in the study	
☒	☐	Antibodies
☒	☐	Eukaryotic cell lines
☒	☐	Palaeontology and archaeology
☒	☐	Animals and other organisms
☒	☐	Human research participants
☒	☐	Clinical data
☒	☐	Dual use research of concern

Methods

n/a	Involved in the study	
☒	☐	ChIP-seq
☐	☒	Flow cytometry
☒	☐	MRI-based neuroimaging

Flow Cytometry

Plots

Confirm that:

☒ The axis labels state the marker and fluorochrome used (e.g. CD4-FITC).

☒ The axis scales are clearly visible. Include numbers along axes only for bottom left plot of group (a 'group' is an analysis of identical markers).

☒ All plots are contour plots with outliers or pseudocolor plots.

☒ A numerical value for number of cells or percentage (with statistics) is provided.

Methodology

Sample preparation	The cells were cultured in OAV-supplemented or glucose-supplemented minimal medium .
Instrument	Amnis™ ImageStream™X imaging flow cytometry
Software	IDEAS software v.6.2.183.0
Cell population abundance	SpeedBead calibration reagents were used for daily calibration as internal beads and run concurrently for real-time velocity detection and autofocusing. Approximately 10,000 cells (data points) were acquired and gated according to the fluorescence intensity and fluorescence aspect ratio intensity to exclude the internal beads.
Gating strategy	Firstly, we made the gating on dot map with fluorescent intensity and fluorescent Aspect ratio intensity in order to exclude the internal size standard beads and fragments of dead cells, and selected the value of fluorescent Aspect ratio intensity more than 0. Furthermore, we made the second gating on the cell distribution in fluorescent gradient RMS to obtain the cell image which was in focus for analyzing the cell properties, and selected the value of fluorescent gradient RMS between 30 and 60.

☒ Tick this box to confirm that a figure exemplifying the gating strategy is provided in the Supplementary Information.
